# P-1371. Identifying Occurrences of Plasmid-Borne Antimicrobial Resistance Gene Transfer between Diverse Bacterial Species within Environmental Biofilms Using Proximity-Ligation Sequencing

**DOI:** 10.1093/ofid/ofae631.1548

**Published:** 2025-01-29

**Authors:** Windy Tanner, Ayesha Tajammul, Scott Benson, Jamil Ahmed, James VanDerslice, William Brazelton

**Affiliations:** University of Utah, Salt Lake City, Utah; Mehran University of Engineering and Technology, Jamshoro, Sindh, Pakistan; University of Utah, Salt Lake City, Utah; Rashid Latif Khan University Medical College, Lahore, Lahore, Punjab, Pakistan; University of Utah, Salt Lake City, Utah; University of Utah, Salt Lake City, Utah

## Abstract

**Background:**

Antimicrobial resistance is a critical global concern. Pathogens may become resistant through acquisition of mobile genetic elements harboring antimicrobial resistance genes (ARGs). Environmental biofilms provide an excellent venue for ARG transfer via mobile elements. Mobile ARG dissemination pathways in the environment require a better understanding. Here we use proximity-ligation (Hi-C) and metagenomic sequencing to pair plasmids and ARGs to their bacterial hosts and present evidence of plasmid sharing between bacterial species.

**Methods:**

Eight environmental biofilm swabs collected from water sources in Sindh Province, Pakistan were equally aliquoted for preparation by 1) DNA cross-linking for proximity ligation and Hi-C metagenomic sequencing, and 2) traditional shotgun metagenomic sequencing. Metagenomic Hi-C was used to analyze the proximity-ligation and shotgun sequencing data to construct metagenomic-assembled genomes, determine if ARGs were chromosomally- or plasmid-located, and associate mobile genetic elements and ARGs with their host cells.

**Results:**

Actinomycetia, Gammaproteobacteria, and Alphaproteobacteria were the most frequently detected taxa in most samples, with Bacteroidia also being at higher numbers than other taxa in most samples. Multiple classes of ARGs in the sample were identified as being located on plasmids, including aminoglycoside ARGs (e.g. aph(3'), aph(6)), beta-lactam RGs (blaCTX-M-15, blaEC, blaTEM-1, blaOKP-A), macrolide RGs (ere(A), erm(X), mph(A), mef(C), mph(G)), quinolone RG qnrS1, sulfonamide RG sul1, and several tetracycline and metal ARGs. TetC, blaA2, and aac(3)I, were only found on bacterial chromosomes. Analysis of unique assembled ARG-bearing plasmids and taxonomic clusters revealed plasmid sharing between bacterial species, some of which were not closely related taxa.

**Conclusion:**

Mobile ARGs are common in environmental biofilms. These ARGs have the potential to spread within biofilms between species, as evidenced by shared plasmid between different bacterial species. The environment should be considered an important source for mobile ARG dissemination between bacterial species.

**Disclosures:**

**All Authors**: No reported disclosures

